# Prevalence and Antimicrobial Resistance of *Campylobacter* Species in Diarrheal Patients in Mymensingh, Bangladesh

**DOI:** 10.1155/2021/9229485

**Published:** 2021-08-03

**Authors:** Md. Ashikur Rahman, Priyanka Rani Paul, Nazmul Hoque, Sk Shaheenur Islam, A. K. M. Ziaul Haque, Mahmudul Hasan Sikder, Aminul Matin, Shinji Yamasaki, S. M. Lutful Kabir

**Affiliations:** ^1^Department of Microbiology and Hygiene, Bangladesh Agricultural University, Mymensingh 2202, Bangladesh; ^2^Department of Pharmacology, Bangladesh Agricultural University, Mymensingh 2202, Bangladesh; ^3^Health Care Center, Bangladesh Agricultural University, Mymensingh 2202, Bangladesh; ^4^Graduate School of Life and Environmental Sciences, Osaka Prefecture University, Osaka 598-8531, Japan

## Abstract

*Campylobacter* enteritis is the leading cause of gastroenteritis in humans worldwide including Bangladesh. The objectives of this study were to estimate the prevalence and antimicrobial-resistance status of *Campylobacter* spp. in human diarrheal samples collected from Surya Kanta Hospital, Mymensingh, Bangladesh. In this study, we evaluated a total of 330 clinical samples for the presence *Campylobacter* spp. via cultural and biochemical tests and molecular assays. Furthermore, antimicrobial susceptibility testing for *Campylobacter* species was accomplished by the standard agar disc diffusion technique against eight commercially available antimicrobial agents. A pretested semistructured questionnaire was used to capture the data on socioanthropological factors from the diarrheal patients. Pearson's chi-square test was performed, and a *p* value of <0.05 was considered for the level of significance. Nearly one in three diarrheal patients admitted in this hospital were infected with *Campylobacter* spp. Overall prevalence of *Campylobacter* spp. was estimated to be 31.5% (104/330) that comprised the prevalence of *C. jejuni*, 21.8% (*n* = 72), and *C. coli*, 9.6% (*n* = 32). Among the positive cases, the prevalence of *Campylobacter* was higher in the age group 0-5 years (52%) followed by 6-18 years (42.7%), 19-40 years (34.0%), 41-60 years (25.4%), and >60 years (10.5%). Age, family level's personal hygiene, and involvement with animal husbandry were captured as potential determinants to be associated with the *Campylobacter* positive status. Among the isolates, 27.3% (*n* = 20) of *C. jejuni* and 31.2% (*n* = 10) of *C. coli* demonstrated as multidrug-resistant (MDR) to three or more antimicrobial agents. The present study shows that *Campylobacter* spp. is most prevalent among the hospital-admitted diarrheal patients, and proper measures should be taken to reduce the burden focusing on the potential determinants.

## 1. Introduction

*Campylobacter* spp. are considered to be zoonotic pathogens that cause foodborne infection throughout the world [1, 2]. These pathogens are the leading causative agents of sporadic bacterial diarrhea worldwide [3, 4]. It was estimated that more than 16 million people got sick with nearly 37 thousand deaths in 2010 worldwide that were connected with *Campylobacter* spp. as a single infection [3]. *Campylobacters* are the most frequently isolated bacterial enteric pathogens both in developed and low- and middle-income countries (LMICs) [5]. In developed countries, *Campylobacter jejuni* and *Campylobacter coli* are the predominately isolated species associated with human bacterial diarrheal disease [6, 7]. Primarily, most of the zoonotic *Campylobacters* are well adapted as a commensal in the gastrointestinal tract of poultry, cattle, sheep, and pigs [8] and can act as reservoirs [9]. Humans can be infected with *Campylobacter* via consumption of contaminated meat and poultry including dairy products [10, 11]. Additionally, *Campylobacter* can contaminate the soil or water bodies to facilitate human infection or even direct human-to-human transmission [12].

*Campylobacter* infections exhibit variable clinical signs from sudden diarrhea to vomition, and abdominal cramps with onset of fever could continue at least for a week [13]. *Campylobacter jejuni* could cause severe chronic symptoms called Guillain–Barré syndrome (GBS) following the infection as a consequence of autoimmune disease [14].

*C. jejuni* is an important etiological agent of childhood diarrhea (25.5%) in Bangladesh [15]. However, the Guillain–Barré syndrome (GBS) which is connected to *Campylobacter* infection causes acute flaccid paralysis (AFP) established in Bangladesh with an estimated incidence rate of 3.25 cases/100,000 children under 15 years of age [16, 17]. Many efforts have been made to minimize *Campylobacter* infection and its associated GBS risks without considering the source of introduction in the LMICs. Therefore, a significant burden of *Campylobacter* prevails in such counties [18], even in Bangladesh.

Earlier studies in Bangladesh reported a variable level of prevalence rate during the 1990s ranging from 17 to 26% with *C. jejuni* [19, 20] and 9.45% with *C. jejuni* and 2.68% with *C. coli* spanning from 2005 to 2008 [21]. A few separate studies confirmed 8.5% prevalence for *Campylobacter jejuni* and *Campylobacter coli* in fecal specimens [22] and 15.3% and 11.3% prevalence of *Campylobacter jejuni* and *Campylobacter coli*, respectively [23]. However, a most recent study confirmed the prevalence of *C. jejuni/coli* as 28.3% in 2019 [24]. Nowadays, the widespread use of antimicrobial agents both in human and veterinary practices is a global concern [25]. This practice appears to be increased quickly in LMICs where rampant use of antibiotics is more common [26]. As a result, the resistance of *Campylobacter* species to antimicrobials has been documented throughout the world [27]. The emergence of antimicrobial-resistant *Campylobacter* species due to the overuse of antimicrobial agents in food animal production enables the spreading of antimicrobial-resistant bacteria [28]. This phenomenon highlights a serious impact on both veterinary and human health regarding food safety and public health issues.

In Bangladesh, several studies have been conducted with very few explored socioanthropological determinants of the occurrence of *Campylobacter* spp. along with antimicrobial-resistant pattern of the isolates from human diarrheal patients. Therefore, the present study was designed to estimate the prevalence and antimicrobial resistance profile along with socioanthropological determinants of *Campylobacter* spp. infection in diarrheal patients from the Surya Kanta Hospital of Mymensingh district in Bangladesh.

## 2. Materials and Methods

### 2.1. Sample Collection and Shipment

Three hundred thirty (*N* = 330) stool specimens were collected randomly from patients suffering from different magnitudes of acute gastroenteritis symptoms including diarrhea admitted to the Surya Kanta Hospital, Mymensingh Medical College, Mymensingh, during the period from June 2019 to June 2020. A team composed of a physician and a lab technician were engaged in sampling and subsequent data collection. Aseptic measures were taken during the collection of samples. A single rectal swab sample was obtained from each patient, stored, and transported in Cary-Blair transport media. A unique identification number was given for each sample and transferred to the laboratory of the Department of Microbiology and Hygiene, Bangladesh Agricultural University (BAU), Mymensingh, maintaining with a cool chain (4–6°C).

### 2.2. Questionnaire Survey

A semistructured interview questionnaire was developed and used for data collection from diarrheal patients on socioanthropological factors: age, sex, educational status, family level personal hygiene, and animal (ruminant, pets, and poultry) husbandry status including food habits. The questionnaire was translated into the local Bengali dialect for the face-to-face interview so that the respondent could easily understand its content.

### 2.3. Isolation and Identification

Isolation and identification of *Campylobacter* spp. were accomplished through the filtration method [29]. In brief, each of the stool specimens was suspended in 500 *μ*l of sterile saline. A portion of 100 *μ*l of sample was spread onto the surface filters of blood agar base no. 2 including Skirrow supplements and permitted to stand for 30 min at room temperature. After 30 min, the filter was removed from the Skirrow blood agar and then the plates were incubated at 37°C for 48 h in microaerophilic condition (5% O_2_, 10% CO_2_, and 85% N_2_). The incubated media were inspected for bacteria growth after 48 h. In Skirrow blood agar media, grey, flat, and irregularly spreading colonies were observed. The colonies were evaluated via Gram's staining, and Gram-negative curves were observed under the light microscope. Gram's stain positive colonies were further utilized in biochemical tests, namely, catalase, hippurate hydrolysis, and oxidase tests. The selected colonies that were found to be positive in Gram's staining and biochemical tests were further subcultured through the same procedure to get a pure colony. Thus, pure isolates obtained were used for further evaluations. Discrimination of *Campylobacter* isolates was completed based on growth characteristics as well as biochemical tests as per the standard protocols [30–32].

### 2.4. Molecular Identification by PCR

Gram's staining and biochemical test positive isolates of the *Campylobacter* growth colony were validated by PCR assay as *Campylobacter* spp. The DNA materials were extracted from the pure colonies as per the procedure by Hoshino et al. [33]. Employing oligonucleotide primers, the genus of *Campylobacter* was confirmed through the amplification of the targeted 16S rRNA gene, as per the method labeled by Samosornsuk et al. [34] (Table 1).

After confirmation of *Campylobacter* spp. via 16S rRNA gene-based PCR assay, a *cdtA* gene-based multiplex PCR was done for the detection of different species of *Campylobacter* (i.e., *C. jejuni*, *C. coli*, and *C. fetus*) as per the protocol described by Linton et al. [36]. In these multiplex PCR assays, *C. jejuni* ATCC 33560, *C. coli* ATCC 33559, and *C. fetus* ATCC 27374 strains in DNA templates were utilized as a positive control. However, *Escherichia coli* ATCC 25922 was utilized as a negative control. In the multiplex PCR assay, for those isolates confirmed as *C. jejuni*, their identity was further substantiated by a marker-based (*hipO* gene) PCR assay [36]. The sequences of the primers and parallel amplicon sizes including annealing temperatures of PCR are shown in Table 1. The PCR products were pictured in gel electrophoresis (1.5% agarose, Invitrogen, Carlsbad, CA, USA), and after coloring with ethidium bromide (0.5 *μ*g ml^−1^) and decoloring with distilled water for 10 min, gel pictures were captured via a UV transilluminator (Biometra, Göttingen, Germany).

### 2.5. Antimicrobial Sensitivity Testing

Isolates of *Campylobacter* spp. were tested via the disk diffusion method [37] using eight (8) commercially available antimicrobials in Bangladesh, viz., amoxicillin (30 *μ*g), ciprofloxacin (5 *μ*g), azithromycin (30 *μ*g), erythromycin (30 *μ*g), tetracycline (30 *μ*g), streptomycin (10 *μ*g), gentamicin (10 *μ*g), and ceftriaxone (30 *μ*g) (HiMedia, Mumbai, India). The zones of inhibition growth were evaluated as the diameter zone as per parameters specified by the Clinical and Laboratory Standard Institute (CLSI) [38], thus established as resistant (R), intermediate resistant (I) or susceptible (S) against the antimicrobial agents. In this testing, the *E. coli* ATCC 25922 strain was used as a quality control organism. All evaluations were validated by conducting at least two replications of the disk diffusion test. Multidrug resistance (MDR) was decided as the resistance to a minimum of three different classes of antimicrobial agents [39].

### 2.6. Data Management and Statistical Analysis

Data on socioanthropological factors and laboratory assessment were captured in Microsoft Excel 2010 (MS Excel) sheets, and data were cleaned and checked for consistency for analysis. The data were analyzed by the Epi Info 7 program [40] both for descriptive and inferential interpretations. Pearson's chi-square test was done to find out the association between determinates and *Campylobacter* infection status with a *p* value of <0.05 taken as statistical significance for all analyses.

### 2.7. Ethics Approval and Consent to Participate

The Ethical Committee of the Bangladesh Agricultural University (BAU) approved this study as a part of the larger project (AWEEC/BAU/2019 (45)). However, a separate permission was obtained from the Director of Mymensingh Medical College Hospital, Mymensingh, Bangladesh (No: 2313 on 8 April 2019) for human screening. Additionally, an oral consent was obtained from each participant as a substantial number of patients included under this study could not read and write.

## 3. Results

### 3.1. Prevalence of *Campylobacter* spp.

Of 330 samples, 104 were confirmed as *Campylobacter* spp. via culture, biochemical tests, and, finally, molecular assay (16S rRNA). All positive *Campylobacter* isolates (104) exhibited a particular amplicon size of 1530 bp through a 16S rRNA gene-specific polymerase chain reaction (PCR) (Supplementary Figure S1-a). Subsequently, a *cdtA* gene-based multiplex PCR was accomplished for the detection of different genus of *Campylobacter*, viz., *C. jejuni*, *C. coli*, and *C. fetus*. In this PCR assay, *C. jejuni* and *C. coli* produced an amplicon size of 631 bp and 329 bp, respectively, as a verifying test for species confirmation (Supplementary Figure S1-b). However, the isolates of *C. jejuni* were further confirmed via a robust marker *hipO* gene-based PCR assay that presented a 735 bp amplicon size (Supplementary Figure S1-c). Thus, the overall prevalence o*f Campylobacter* was confirmed as 31.5% (104/330) that represented 21.8% (72/330) and 9.7% (32/330) prevalence for *C. jejuni* and *C. coli*, respectively (Table 2). In this study, amongst 104 isolates, 69.2% and 30.8% were confirmed as *C. jejuni* and *C. coli*, respectively.

### 3.2. Distribution of Prevalence among Different Determinants

**Figure 1:**
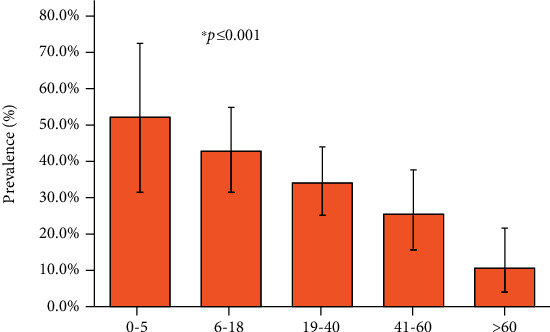
Distribution of prevalence with 95% confidence interval (CI) at different age groups in Surya Kanta Hospital, Mymensingh, from June 2019 to June 2020. The prevalence among the different age groups was found to be statistically significant from each other (^∗^*p* ≤ 0.001).

A higher prevalence was observed in female (35.2%) patients than in male (29.3%) patients. However, there is no statistical significance among the sexes. Considering the age group, the higher prevalence (52%) was observed in the age group 0-5 years, followed by 6-18 years (42.7%), 19-40 years (34.0%), 41-60 years (25.4%), and >60 years (10.5%) (Figure 1 and Table 3).

A lower level of *Campylobacter* prevalence (21.5%) was documented in good hygienic practice patients and found to be statistically significant. Similarly, involvement of livestock (cattle, sheep, goat, and poultry) rearing was found to be risky practices as higher prevalence (43.2%) was recognized. Similarly, regarding food habits, *Campylobacter* was found to be more prevalent in those patients who ate home-prepared food than the patients who ate other sources of food. However, no statistical significance was found (Table 2).

### 3.3. Antibiogram

#### 3.3.1. Antimicrobial Susceptibility Testing

In this study, of 72 isolates of *C. jejuni*, 54.2% (*n* = 39) were found to be susceptible to azithromycin, followed by streptomycin (51.4%, *n* = 37), gentamycin and/or ceftriaxone (47.2%, *n* = 34), ciprofloxacin (44.4%, *n* = 32), tetracycline (40.3%, *n* = 29), erythromycin (33.4%, *n* = 24), and amoxicillin (20.8%, *n* = 15). However, 34.7% (*n* = 25) isolates exhibited intermediate susceptibility to gentamycin and/or streptomycin and 30.6% (*n* = 22) isolates to azithromycin and/or ceftriaxone. On the contrary, 32 isolates of *C. coli* 62.5% (*n* = 20) were found susceptible to ceftriaxone and/or ciprofloxacin and streptomycin followed by gentamycin (37.5%, *n* = 12), azithromycin (28.1%, *n* = 9), tetracycline (21.9%, *n* = 7), and amoxicillin (18.8%, *n* = 6). Conversely, 40.6% (*n* = 13) isolates were shown intermediately susceptible to azithromycin followed by streptomycin (28.1%, *n* = 9) and tetracycline (18.8%, *n* = 6) (Table 4).

#### 3.3.2. Antimicrobial-Resistant Status

Among the 72 isolates of *C. jejuni*, 72.3% (*n* = 52) were presented as resistant against 1-2 antimicrobial agents that comprised 33.4% (*n* = 24) isolates to single antimicrobial agents (AMX, ERY) and 38.9% (*n* = 28) to two antimicrobial agents (AMX-TET and AMX-STR). Similarly, of 32 isolates of *C. coli*, 68.7% (*n* = 22) were presented as resistant against 1-2 antimicrobial agents that comprised 50.0% (*n* = 16) isolates to single antimicrobial agents (AMX, ERY) and 18.7% (*n* = 6) to two antimicrobial agents (AMX-TET and AMX-STR).

**Figure 2:**
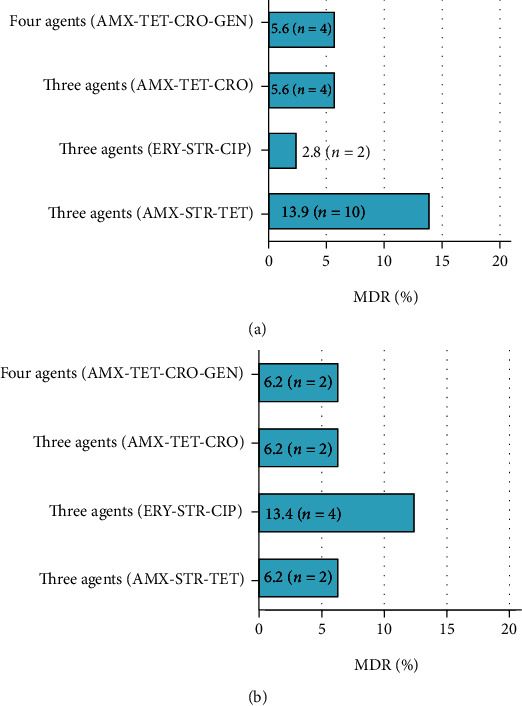
Multidrug-resistant (MDR) status of (a) *C. jejuni* isolates (*n* = 72) presented resistant to 3 agents (AMX-STR-TET) (*n* = 10, 13.9%), 3 agents (ERY-STR-CIP) (*n* = 2, 2.8%), 3 agents (AMX-TET-CRO) (*n* = 4, 5.6%), and 4 agents (AMX-TET-CRO-GEN) (*n* = 4, 5.6%); (b) *C. coli* isolates (*n* = 32) presented resistant to 3 agents (AMX-STR-TET) (*n* = 2, 6.2%), 3 agents (ERY-STR-CIP) (*n* = 4, 12.4%), 3 agent (AMX-TET-CRO) (*n* = 2, 6.2%), and 4 agents (AMX-TET-CRO-GEN) (*n* = 2, 6.2%) at Surya Kanta Hospital, Mymensingh, from June 2019 to June 2020.

In this study, among the isolates of *C. jejuni* (*N* = 72), 10 (13.9%), 2 (2.8%), and 4 (5.6%) were found to be multidrug-resistant against three antimicrobial agents, namely, AMX-STR-TET, ERY-STR-CIP, and AMX-TET-CRO, respectively. However, 4 (5.56%) isolates were documented to be resistant against 4 antimicrobial agents (AMX-TET-CRO-GEN) (Figure 2(a)).

Correspondingly, among the isolates of *C. coli* (*N* = 32), 2 (6.2%), 4 (12.4%), and 2 (6.2%) were found to be multidrug-resistant against three antimicrobial agents, viz., AMX-STR-TET, ERY-STR-CIP, and AMX-TET-ERY, respectively. Nevertheless, 2 (6.3%) isolates were shown resistant against 4 antimicrobial agents (AMX-TET-CRO-GEN) in this study (Figure 2(b)).

## 4. Discussion

We evaluated the prevalence, risk factors, and antimicrobial resistant status of *Campylobacter* spp. in diarrheal specimens collected from the patients admitted in Surya Kanta Hospital, Mymensingh, Bangladesh. The study confirmed that nearly one-third of the patients (31.5%) was found to be positive with *Campylobacter* spp., of which *Campylobacter jejuni* (21.8%) was captured as the key contributor of human bacterial diarrhea. The finding of this study is in accordance to similar studies conducted in Bangladesh [15, 19–23].

In this study, the prevalence of *Campylobacter* spp. was found higher in females than the males but not statistically significant. In Bangladesh, females are more likely to be at an increased risk of *Campylobacter* exposure as they are involved with household livestock rearing (cattle, sheep, goat, and poultry) than males. This finding conforms to the result of an earlier study conducted in Bangladesh [23]. However, improved personal hygiene practices would contribute towards the lower prevalence in males.

The distribution of prevalence of *Campylobacter* spp. was found to be significantly varied among the different age groups (*p* ≤ 0.001). In this study, the higher prevalence was observed in the youngest age group (0-5 years). Earlier studies also reported variable prevalence of *Campylobacter* as 25.5% [15] and 12.9% [41] but consistent with the most susceptible young patients. *Campylobacter* can cause infection in all age groups; however, the clinical presentation may differ by different age groups of patients established in previous studies [23, 42–44]. Children at a young age are not aware on hygiene and sanitation practices. Commonly, in the rural settings of Bangladesh, farmers keep the livestock close to their house which offers regular contact of young children with the animals which is likely to further the risk of exposure of infection.

Family-level personal hygiene and involvement with livestock rearing were found to be associated with a higher level of *Campylobacter* exposure. The main routes of transmission of *Campylobacter* are the consumption of poultry and involvement with poultry rearing activities [45] or exposures resulting from environmental contamination by cattle manure [46, 47] that facilitate potential introduction in humans through the food chain [48]. However, exposure to livestock was documented as a significant association with higher *Campylobacter* positivity status [49]. Therefore, family-level involvement with livestock rearing including poor personal hygiene was presented to be a higher level of *Campylobacter* occurrence in this study.

Our study found that 20.8 to 51.4% and 15.6 to 62.5% isolates of *C. jejuni* (*n* = 72) and *C. coli* (*n* = 30), respectively, were susceptible to all antimicrobial agents. The isolates *C. jejuni* and *C. coli* exhibited resistant to amoxicillin (54.2%), erythromycin (45.8%), tetracycline (43%), ciprofloxacin (33.3%), and azithromycin (22.2%) and amoxicillin (68.7%), erythromycin (62.5%), tetracycline (59.3%), and ciprofloxacin and/or azithromycin (31.5%), respectively. This finding is narrowly corroborated by Albert et al. [19] in Bangladesh as *Campylobacter* isolates show resistance to ciprofloxacin that varies from 65 to 88% during 2005 to 2008 [21]. However, the finding of this study is consistent with a similar study conducted in diarrheal patients in Finland [50]. However, antimicrobial resistance was documented as 46.7%, 35.6%, and 17.8% in *C. jejuni*, *C. coli*, and *C. upsaliensis* isolates, respectively, in beef cattle of South Africa [51]. Conversely, higher levels of resistance to ciprofloxacin and tetracycline as >80% and> 70%, respectively, were captured in the diarrheal sample in the Arabian Gulf region [52].

Our study demonstrated that 27.8% (*n* = 20) and 31.2% (*n* = 10) of *C. jejuni* and *C. coli*, respectively, showed as multidrug resistance to 3 or more antimicrobial drugs, consistent with similar studies [23, 50, 53–55]. The MDR noticed in this survey is likely to be a potential reflection of imprudent use of antimicrobials due to their easy accessibility at local drug stores throughout the country [21].

Based on the study findings, risk reduction measures to be taken to address health education for the household livestock keepers, personal hygiene, microbiological evaluation of the isolates through culture and molecular assessment, and prudent use antimicrobials are needed.

## 5. Conclusions

The present study shows occurrence of *Campylobacter* infection in diarrheal patients, of which *Campylobacter jejuni* was captured as an abundant species. The higher frequency was observed in 0-5 years age, family-level poor hygienic practices, and involvement of animal husbandry. Therefore, activities on awareness creating behavioral change relating to personal hygiene like hand washing and sanitization after using the toilet or animal contact are needed. Children 0-5 years of age should avoid contact with livestock. The treatment of diarrhea patients should be based on an updated database on the susceptibility status of *Campylobacter* spp. instead of clinical signs. A physician should consider that diagnosing diarrhea infection with *Campylobacter* spp. is crucial among the other enteric pathogens. Cognizing the burden of *Campylobacter* diarrheal disease is significant for framing effective control programs targeting the overall decline of diarrheal disease in all ages of people. Further investigations are required to substantiate the role of domestic animals in the spreading of *Campylobacter* spp. including species diversities/serotyping.

## Figures and Tables

**Table 1:** Primers and conditions used for different PCR assays.

Primers	Sequence (5′-3′)	Target/purpose	Amplicon size (bp)	PCR condition (30 cycle)			Reference
				Denaturation	Annealing	Extension	
16S9F 16S1540R	GAGTTTGATCCTGGCTC AAGGAGGTGATCCAGCC	16S rRNA	1530	94°C, 30 s	47°C, 30 s	72°C, 90 s	[34]
Cj-CdtAU2 Cj-CdtAR2	AGGACTTGAACCTACTTTTC AGGTGGAGTAGTTAAAAACC	*CjcdtA*	631	94°C, 30 s	53°C, 30 s	72°C, 30 s	[35]
Cc-CdtAU1 Cc-CdtAR1	ATTGCCAAGGCTAAAATCTC GATAAAGTCTCCAAAACTGC	*CccdtA*	329				
Cf-CdtCU2 Cf-CdtCR2	AACGACAAATGTAAGCACTC TATTTATGCAAGTCGTGCGA	*CfcdtA*	489				
HIP400F HIP1134R	GAAGAGGGTTTGGGTGGTG AGCTAGCTTCGCATAATAACTTG	*hipO* gene	735	94°C, 30 s	55°C, 30 s	72°C, 45 s	[36]

**Table 2:** Distribution of *Campylobacter* isolates in human diarrheal samples (*N* = 330) at Surya Kanta Hospital, Mymensingh, from June 2019 to June 2020.

Parameter/variable	Positive sample (*n*)/number of sample tested (*N*)	Prevalence (%) with 95% CI
*Campylobacter* spp.	104/330	31.5 (26.5-36.8)
*Campylobacter jejuni* (*n* = 72)	72/330	21.8 (17.5-26.7)
*Campylobacter coli* (*n* = 32)	32/330	9.7 (6.7-13.4)

*N*: total sample tested; *n*: number of positive isolates; CI: confidence interval.

**Table 3:** Distribution of *Campylobacter* spp. among different determinants of diarrheal patients (*N* = 330) at Surya Kanta Hospital, Mymensingh, from June 2019 to June 2020.

Determinants (*n*)	Positive	Prevalence (%) with 95% CI	Pearson's chi-square *p* value
Sex			
Male (*n* = 208)	61	29.3 (23.2-26.0)	0.26
Female (*n* = 122)	43	35.3 (26.8-44.4)	
Age (years)			
0-5 (*n* = 25)	13	52.0 (31.3-72.2)	≤0.001
6-18 (*n* = 75)	32	42.7 (31.3-54.7)	
19-40 (*n* = 106)	36	34.0 (25.0-43.8)	
41-60 (*n* = 67)	17	25.4 (15.5-37.5)	
>60 (*n* = 57)	6	10.5 (4.0-21.5)	
Education status			
Illiterate/not applicable	56	37.7 (26.6-41.5)	0.38
Literate	48	29.3 (22.4-36.9)	
Family-level personal hygiene			
Good (*n* = 228)	49	21.5 (16.3-27.4)	≤0.001
Bad (*n* = 102)	55	53.9 (43.8-63.8)	
Involvement with household livestock rearing			
Yes (*n* = 132)	57	43.2 (34.6-52.1)	0.001
No (*n* = 198)	47	23.7 (18.0-30.3)	
Food habit			
Home prepared (*n* = 227)	77	33.9 (27.8-40.5)	0.16
Other (fast food/food from restaurant) (*n* = 103)	27	26.2 (18.0-35.8)	

CI: confidence interval.

**Table 4:** Antimicrobial susceptibility pattern of *Campylobacter jejuni* (*n* = 72) and *Campylobacter coli* (*n* = 32) identified by the disk diffusion method at Surya Kanta Hospital, Mymensingh, from June 2019 to June 2020.

Antimicrobial agents	Susceptible (%, *n*) rate of isolates by species		Intermediate (%, *n*) rate of isolates by species		Resistant (%, *n*) rate of isolates by species	
	*C. jejuni*	*C. coli*	*C. jejuni*	*C. coli*	*C. jejuni*	*C. coli*
Amoxicillin (AMX)	20.8 (15)	18.8 (6)	25 (18)	12.5 (4)	54.2 (39)	68.7 (22)
Tetracycline (TET)	40.3 (29)	21.9 (7)	16.7 (12)	18.8 (6)	43 (31)	59.3 (19)
Gentamicin (GEN)	47.2 (34)	37.5 (12)	34.7 (25)	12.5 (4)	18.1 (13)	50 (16)
Streptomycin (ST)	51.4 (37)	62.5 (20)	34.7 (25)	28.1 (9)	13.9 (10)	9.4 (3)
Erythromycin (ERY)	33.4 (24)	15.6 (5)	20.8 (15)	21.9 (7)	45.8 (33)	62.5 (20)
Azithromycin (AZM)	54.2 (39)	28.1 (9)	30.6 (22)	40.6 (13)	15.2 (11)	31.3 (10)
Ciprofloxacin (CIP)	44.4 (32)	62.5 (20)	22.3 (16)	6.3 (2)	33.3 (24)	31.2 (10)
Ceftriaxone (CRO)	47.2 (34)	62.5 (20)	30.6 (22)	6.3 (2)	22.2 (16)	31.2 (10)

*n*: number of isolates; %: percentage.

## Data Availability

The tables, figures and texts in this research article contain the data that support the study conclusions.

## Supplementary Materials

Supplementary Materials Supplementary Figure S1: molecular detection: (a) confirmation of *Campylobacter* spp. by the 16S rRNA gene, lanes 1 and 12: 100 bp DNA ladder (Promega, USA), lanes 2–9: representative positive isolates, lane 10: positive control (*C. jejuni* ATCC 33560), and lane 11: negative control (*Escherichia coli* ATCC 25922); (b) confirmation of *C. jejuni* and *C. coli* via *cdtA* gene-based multiplex PCR assay, lanes 1 and 12: 100 bp DNA ladder (Promega, USA), lanes 2–4: representative positive isolates (*C. coli*), lanes 5–7: representative positive isolates (*C. jejuni*), lane 8: positive control (*C. fetus* ATCC 27374), lane 9: positive control (*C. coli* ATCC 33559), lane 10: positive control (*C. jejuni* ATCC 33560), and lane 11: negative control (*Escherichia coli* ATCC 25922); (c) validation of *C. jejuni* by hippuricase (*hipO*) gene-based PCR, lanes 1 and 12: 100 bp DNA ladder (Promega, USA), lanes 2–9: representative positive isolates, lane 10: positive control (*C. jejuni* ATCC 33560), and lane 11: negative control (*Escherichia coli* ATCC 25922).
