# Supplementary material for: Prevalence and Antimicrobial Resistance of Campylobacter Species in Diarrheal Patients in Mymensingh, Bangladesh
Source: Biomed Res Int. 2021 Aug 3;2021:9229485. doi: 10.1155/2021/9229485 (PMC8357465; doi:10.1155/2021/9229485)
Supplement: Supplementary Materials — Supplementary Figure S1: molecular detection: (a) confirmation of Campylobacter spp. by the 16S rRNA gene, lanes 1 and 12: 100 bp DNA ladder (Promega, USA), lanes 2–9: representative positive isolates, lane 10: positive control (C. jejuni ATCC 33560), and lane 11: negative control (Escherichia coli ATCC 25922); (b) confirmation of C. jejuni and C. coli via cdtA gene-based multiplex PCR assay, lanes 1 and 12: 100 bp DNA ladder (Promega, USA), lanes 2–4: representative positive isolates (C. coli), lanes 5–7: representative positive isolates (C. jejuni), lane 8: positive control (C. fetus ATCC 27374), lane 9: positive control (C. coli ATCC 33559), lane 10: positive control (C. jejuni ATCC 33560), and lane 11: negative control (Escherichia coli ATCC 25922); (c) validation of C. jejuni by hippuricase (hipO) gene-based PCR, lanes 1 and 12: 100 bp DNA ladder (Promega, USA), lanes 2–9: representative positive isolates, lane 10: positive control (C. jejuni ATCC 33560), and lane 11: negative control (Escherichia coli ATCC 25922). [file 9229485.f1.docx]

**Supplementary File Legends**

**Supplementary Figure S1.** Molecular detection: (a) confirmation of *Campylobacter* spp. by 16S rRNA gene, lanes 1 and 12: 100 bp DNA ladder (Promega, USA), lanes 2–9: representative positive isolates, lane 10: positive control (*C. jejuni* ATCC 33560), and lane 11: negative control (*Escherichia coli* ATCC 25922); (b) confirmation of *C. jejuni* and *C. coli* via *cdtA* gene-based multiplex PCR assay, lanes 1 and 12: 100 bp DNA ladder (Promega, USA), lanes 2–4: representative positive isolates (*C. coli*), lanes 5–7: representative positive isolates (*C. jejuni*), lane 8: positive control (*C. fetus* ATCC 27374), lane 9: positive control (*C. coli* ATCC 33559), lane 10: positive control (*C. jejuni* ATCC 33560), and lane 11: negative control (*Escherichia coli* ATCC 25922) and (c) validation of *C. jejuni* by hippuricase (*hipO*) gene-based PCR, lanes 1 and 12: 100 bp DNA ladder (Promega, USA), lanes 2–9: representative positive isolates, lane 10: positive control (*C. jejuni* ATCC 33560), lane 11: negative control (*Escherichia coli* ATCC 25922).


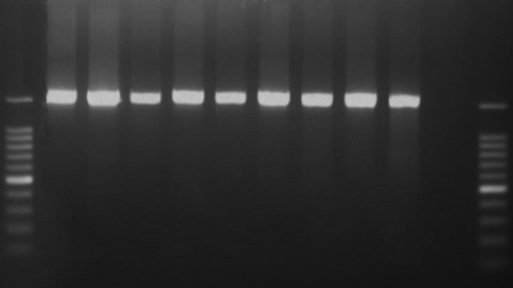


**1 2 3 4 5 6 7 8 9 10 11 12**

**500 bp**

**1000 bp**

**1500 bp**

**500 bp**

**1000 bp**

**1500 bp**

**(a)**


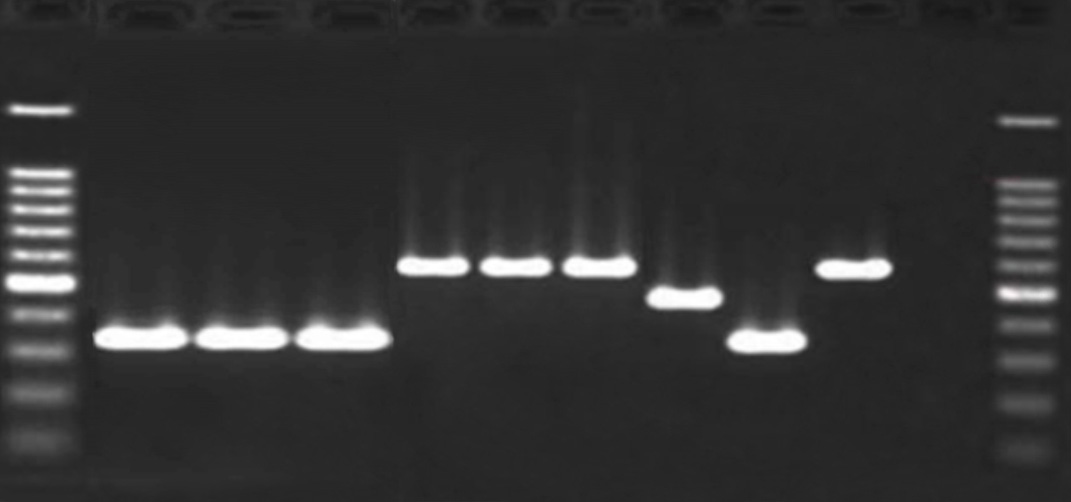


**1 2 3 4 5 6 7 8 9 10 11 12**

**1500 bp**

**1000 bp**

**500 bp**

**500 bp**

**1500 bp**

**1000 bp**

**329 bp**

**489 bp**

**631 bp**

**(b)**


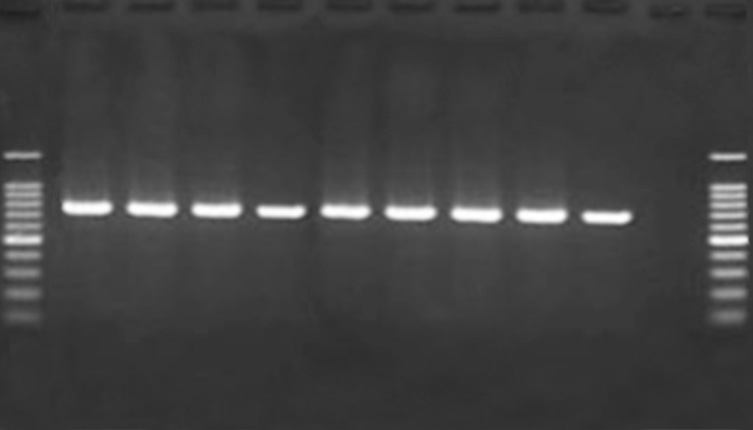


**(c)**

**1 2 3 4 5 6 7 8 9 10 11 12**

**500 bp**

**1000 bp**

**1500 bp**

**500 bp**

**1000 bp**

**1500 bp**
